# Prognostic significance of diabetes mellitus in patients with atrial fibrillation

**DOI:** 10.1186/s12933-021-01232-7

**Published:** 2021-02-11

**Authors:** Andreas S. Papazoglou, Anastasios Kartas, Athanasios Samaras, Ioannis Vouloagkas, Eleni Vrana, Dimitrios V. Moysidis, Evangelos Akrivos, Georgios Kotzampasis, Amalia Baroutidou, Anastasios Papanastasiou, Evangelos Liampas, Michail Botis, Efstratios Karagiannidis, Nikolaos Stalikas, Haralambos Karvounis, Apostolos Tzikas, George Giannakoulas

**Affiliations:** 1 First Department of Cardiology, AHEPA University Hospital, Aristotle University of Thessaloniki, St. Kiriakidi 1, 54636 Thessaloniki, Greece; 2 Laboratory of Computing, Medical Informatics and Biomedical Imaging Technologies, Medical School, Aristotle University of Thessaloniki, Thessaloniki, Greece; 3 Interbalkan European Medical Center, Asklipiou 10, Pylaia, Thessaloniki, Greece

**Keywords:** Atrial fibrillation, Diabetes mellitus, Glycated hemoglobin, HbA1c, Stroke, Mortality

## Abstract

**Background:**

There are limited data on the association of diabetes mellitus (DM) and levels of glycated hemoglobin (HbA1c) with outcomes in patients with atrial fibrillation (AF).

**Methods:**

This retrospective cohort study included patients who were recently hospitalized with a primary or secondary diagnosis of AF from December 2015 through June 2018. Kaplan–Meier curves and Cox-regression adjusted hazard ratios (aHR) were calculated for the primary outcome of all-cause mortality and for the secondary outcomes of cardiovascular (CV) mortality and the composite outcome of CV death or hospitalization. Competing-risk regression analyses were performed to calculate the cumulative risk of stroke, major bleeding, AF- or HF-hospitalizations adjusted for the competing risk of all-cause death. Spline curve models were fitted to investigate associations of HbA1c values and mortality among patients with AF and DM.

**Results:**

In total 1109 AF patients were included, of whom 373 (33.6%) had DM. During a median follow-up of 2.6 years, 414 (37.3%) patients died. The presence of DM was associated with a higher risk of all-cause mortality (aHR = 1.40 95% confidence intervals [CI] 1.11–1.75), CV mortality (aHR = 1.39, 95% CI 1.07–1.81), sudden cardiac death (aHR = 1.73, 95% CI 1.19–2.52), stroke (aHR = 1.87, 95% CI 1.01–3.45) and the composite outcome of hospitalization or CV death (aHR = 1.27, 95% CI 1.06–1.53). In AF patients with comorbid DM, the spline curves showed a positive linear association between HbA1c levels and outcomes, with values 7.6–8.2% being independent predictors of increased all-cause mortality, and values < 6.2% predicting significantly decreased all-cause and CV mortality.

**Conclusions:**

The presence of DM on top of AF was associated with substantially increased risk for all-cause or CV mortality, sudden cardiac death and excess morbidity. HbA1c levels lower than 6.2% were independently related to better survival rates suggesting that optimal DM control could be associated with better clinical outcomes in AF patients with DM.

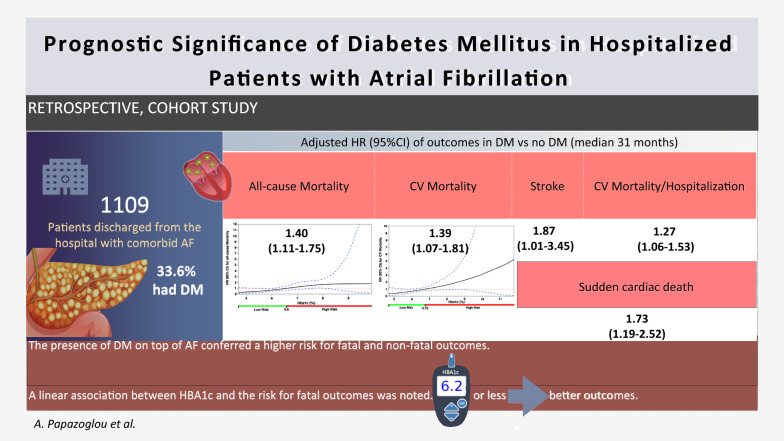

## Background

Both atrial fibrillation (AF) and diabetes mellitus (DM) are medical conditions that nowadays affect western populations at an epidemic rate. These diseases have both evolved into a severe health threat and a costly global health burden. AF is the most clinically important cardiac rhythm disorder; its prevalence will have risen to 16 million by 2050 [1, 2]. At the same time, individuals with DM have approximately 40% greater risk for AF than their non-diabetic counterparts [3]. Well-documented cardiovascular (CV) risk factors put individuals at risk for developing both AF and DM, even if the precise etiology of this relation has long eluded our understanding.

Over the years, numerous studies have examined the influence that DM exercises over the prognosis of AF and over the efficacy of its treatment [4–7]. Nevertheless, the relation between AF and DM still remains a promising field of study, because of the growing evidence that their concomitance affects and perplexes clinical outcomes. Despite the plethora of studies on AF and DM, there is still no sufficient data on the blood glucose regulation as a prognostic modifier in DM patients with AF.

In this study, we analyzed data from a single-center cohort of patients that were hospitalized with a primary or secondary diagnosis of AF in a tertiary academic hospital. We examined the association of DM and blood glucose regulation, as mirrored by the levels of glycated hemoglobin, with the clinical outcomes of patients with AF.

## Methods

### Study design

This is an ancillary study to MISOAC-AF trial (Motivational Interviewing to Support Oral AntiCoagulation Adherence in patients with non-valvular Atrial Fibrillation, ClinicalTrials.gov identifier: NCT02941978) focusing on patients with AF and DM. The design and main results of the MISOAC-AF registry have been previously published [8, 9]. In brief, MISOAC-AF demonstrated the impact of a patient-centered interview on improving patients’ adherence to oral anticoagulation (OAC) treatment.

The study protocol was approved by the Institutional Review Board of Aristotle University of Thessaloniki, and the trial was conducted in compliance with the principles of the Declaration of Helsinki [10].

### Data sources

The data used in our study were extracted from the MISOAC-AF database for the time range of December 2015 to April 2020. Medical history, baseline clinical characteristics, laboratory and echocardiographic data prior to the date of hospital discharge, as well as discharge diagnoses and follow-up data on clinical outcomes were all included in the database. All data were collected by trained independent investigators after obtaining patients’ written informed consent.

### Study population

The study comprised adult patients, consecutively enrolled in the prospective cohort of the MISOAC-AF trial. Comorbidity with AF was the shared feature of all patients included, irrespective of the discharge diagnoses from the Cardiology Department of AHEPA Hospital of Thessaloniki, Greece. The presence of end-stage disease not permitting the patients’ follow-up, as well as the absence of data (unavailable or unknown) concerning DM status of patients were the exclusion criteria for the present study.

### Outcomes

All-cause death, i.e. death from any cause, constituted the primary outcome, whereas CV death and specific causes of CV death [cardiac arrest, heart failure (HF)-related death, pulmonary embolism, stroke, hemorrhage], hospitalizations related to AF or HF, stroke and major bleeding episodes, as well as the composite outcome of hospitalization or CV death during the follow-up period were the secondary outcomes. Information was acquired by reviewing discharge letters and through telephonic or in-person interviews with the study participants. The vital status of all patients was additionally verified through the Greek Civil Registration System. A group of blinded to patient randomization individuals were responsible for the adjudication of all events investigating all available follow-up sources.

### Definition of covariates

Patients with DM were defined as those who met at least one of the following criteria: (1) administration of oral anti-diabetic or insulin medication; (2) diagnostic code of DM (International Classification of Diseases-11) in at least one hospital admission; (3) verification of a previous physician-assigned DM diagnosis by study personnel with access to patients’ medical record. AF was defined as previously recorded in medical history or new-onset AF occurring during hospitalization. The latter was recorded as irregular heart rhythm for more than 30 s, without detectable P waves, by performing either a 12-lead electrocardiogram or a 24-h Holter monitor.

### Statistical analysis

Categorical variables are presented as frequencies with percentages, while continuous ones as means with standard deviations. The Pearson Chi square or Fisher’s exact test were used for categorical variables, whereas continuous variables were compared using the Wilcoxon rank-sum or Student’s t-test, depending on the normality of data distributions. The Kaplan–Meier curves were traced for illustrating time-to-event outcomes (e.g. patients censored in the event of death) between patients with and without DM, applying the log rank test for the comparison of the results. Univariable analysis identified significant predictors of each outcome among the clinically relevant parameters. The Cox regression hazard model was developed by forcing clinically relevant variables, univariably associated with each endpoint, into the multivariate analysis. These covariates included: age, body mass index (BMI), prior stroke, coronary artery disease or prior coronary revascularization procedure, renal function, AF subtype (first-diagnosed, paroxysmal, persistent or permanent), use of OAC, angiotensin converting enzyme inhibitors or angiotensin II receptor blockers (ACEI-ARB) and rate control medication after discharge. On our analysis we considered the group of patients without DM as the reference category.

To calculate the risk of the secondary outcomes (stroke, major bleeding, AF- and HF- hospitalizations) during follow-up, a modified Cox regression analysis as described by Fine and Gray was utilized in a setting, accounting for the competing risk of all-cause death [11]. The adjusted hazard ratios (aHR) and subdistribution hazard ratios (extracted from competing-risks regression analyses) are presented with the respective 95% confidence intervals (CI). Moreover, in order to assess the relationship between continuous HbA1c levels and each outcome, we performed a spline curve analysis. These spline curves display the adjusted hazard ratios for the outcome on the y-axis versus the HbA1c blood levels on the x-axis, whereas dashed lines represent 95% confidence intervals. Following backward regression of candidate confounding variables, the hazard ratios were adjusted for age, gender, BMI, history of dyslipidemia, smoking history, alcohol consumption history, N-terminal pro b-type natriuretic peptide (NT-proBNP), estimated glomerular filtration rate (eGFR), and hemoglobin stages at discharge.

All analyses were conducted with the SPSS Statistics for Windows, Version 26.0 (Armonk, NY: IBM Corp) and Stata statistical software, release 13 (StataCorp) and a two-sided p value of less than 0.05 was considered statistically significant.

## Results

### Baseline characteristics

A total of 1140 patients were registered in our database, out of which 1109 were analyzed and 31 were omitted due to incomplete data on DM status. Comorbid DM was present in 373 (33.6%) patients.

Baseline clinical and demographic characteristics of the study participants by diabetes status are given in Table 1. Patients with DM had higher age and BMI than non-DM counterparts. Furthermore, DM was significantly associated with the prevalence of other medical conditions, such as hypertension, dyslipidemia, chronic obstructive pulmonary disease, HF, stroke, myocardial infarction, vascular and chronic kidney disease.

**Table 1:** Baseline characteristics and comorbidities in AF patients with and without DM

Clinical characteristics	Total population (n = 1109)	AF with DM (n = 373)	AF without DM (n = 736)	P value (comparing DM vs no-DM)
Gender	M: 603 F: 506	M:196 F: 177	M: 407 F: 329	*0.350*
Age (years)	73.6 ± 10.9	75.2 ± 8.8	72.9 ± 11.7	*< 0.001*
Body mass index (kg/m^2^)	28.5 ± 5.4	29.5 ± 5.6	28.0 ± 5.4	*< 0.001*
Glomerular filtration rate (GFR) by CKD-EPI (mL/min/1.73 m^2^)	60.4 ± 23.6	57.0 ± 22.4	64.7 ± 23.6	*< 0.001*
Left ventricular ejection fraction (%)	49.2 ± 12.2	48.4 ± 12.3	49.7 ± 12.1	*0.113*
NT-pro-BNP	2430.2 ± 5525.3	2331.5 ± 4991.1	2289.5 ± 5460.2	*0.915*
Glycated hemoglobin (%)	6.52 ± 1.10	6.85 ± 1.14	5.77 ± 0.43*	*< 0.001*
Medical histories	N (%)			
Hypertension	888 (80.1)	317 (87.3)	571 (78.0)	*< 0.001*
Dyslipidemia	527 (47.5)	223 (61.4)	304 (41.5)	*< 0.001*
Chronic obstructive pulmonary disease	142 (12.8)	59 (16.2)	83 (11.3)	*0.028*
Heart failure	553 (49.9)	213 (58.8)	340 (46.5)	*< 0.001*
Strokes	164 (14.8)	73 (20.1)	91 (12.5)	*0.001*
Myocardial infarction	228 (20.6)	91 (25.1)	137 (18.7)	*0.018*
Chronic kidney disease	162 (14.6)	84 (23.1)	78 (10.7)	*< 0.001*
Vascular disease	509 (45.9)	210 (57.9)	299 (40.8)	*< 0.001*
Treatment and risk stratification scores				
Rhythm control treatment	409 (36.9%)	109 (29.2%)	300 (40.7%)	*0.002*
Use of antiplatelet(s) at discharge	218 (19.7%)	97 (31.5%)	121 (15.8%)	*0.002*
Use of oral anticoagulant(s) at discharge:				*0.672*
Vitamin K antagonist	282 (25.4%)	101 (27.1%)	181 (24.6%)	
NOAC	522 (47.1%)	171 (45.8%)	351 (47.7%)	
HAS-BLED score at discharge	1.7 ± 1	2 ± 1.1	1.7 ± 1	*< 0.001*
CHA(2)DS(2)-VASc score at discharge	4.4 ± 1.9	5.6 ± 1.6	3.8 ± 1.8	*< 0.001*

Significant p-values are marked in italics

*Glycated hemoglobin (HbA1c) baseline value was documented only in 122/736 (16.6%) AF patients without DM participating in our study

AF: atrial fibrillation; DM: diabetes mellitus; CKD-EPI: Chronic Kidney Disease Epidemiology Collaboration equation; NT-pro-BNP: N-terminal pro b-type natriuretic peptide; NOAC: Non-Vitamin K oral anticoagulant; HAS-BLED: Hypertension, Abnormal renal/liver function, Stroke, Bleeding history or predisposition, Labile International Normalized Ratio, Elderly, Drugs/alcohol concomitantly; CHA_2_DS_2_-VASc: Congestive heart failure, Hypertension, Age ≥ 75 years, Diabetes mellitus, Stroke, Vascular disease, Age 65–74 years, Sex category

Overall, the most prominent AF type was persistent or permanent AF, afflicting more than half of the population (52.5% of diabetic patients and 50% of non-diabetic patients). The presence of DM was associated with a significant increase in risk stratification CHA_2_DS_2_-VASc score [Congestive heart failure, Hypertension, Age ≥ 75 years, Diabetes mellitus, Stroke, Vascular disease, Age 65–74 years, Sex category]-since it is included in the score per se-, as well as HAS-BLED score (Hypertension, Abnormal renal/liver function, Stroke, Bleeding history or predisposition, Labile International Normalized Ratio, Elderly, Drugs/alcohol concomitantly) [12] (both p values < 0.001).

### Clinical outcomes

During a median follow-up of 2.6 years, 414 (36.3%) patients died (Table 2). In particular, 73.3% of deaths were attributed to CV cause, while the presence of DM was significantly associated with higher prevalence of CV death (34.9% versus 23.5%, p < 0.001). In both diabetics and non-diabetics, the most common cause of death was cardiac arrest (DM: 19.3%, non-DM: 10.9%, p < 0.001). HF-related death was the second more common cause of mortality (DM: 13.4%, non-DM: 9.6%, p = 0.058).

**Table 2:** Follow-up outcomes by presence of DM with corresponding HR

Outcome	DM	Non-DM	Adjusted HR* (95% CI)
All-cause death	171/373 (45.8%)	243/736 (33%)	*1.40 (1.11–1.75)*
CV-death	130/373 (34.9%)	173/736 (23.5%)	*1.39 (1.07–1.81)*
Sudden cardiac death	72/373 (19.3%)	80/736 (10.9%)	*1.73 (1.19–2.52)*
HF-realetd death	50/373 (13.4%)	71/736 (9.6%)	1.28 (0.86–1.91)
Major bleeding**	18/340 (5.3%)	29/644 (4.5%)	1.50 (0.78–2.87)
Stroke**	24/340 (7.1%)	28/645 (4.3%)	*1.87(1.01–3.45)*
AF-related hospitalization**	59/340 (17.4%)	115/645 (17.8%)	1.17 (0.82–1.66)
HF-related hospitalization**	35/333 (10.5%)	46/640 (7.2%)	1.40 (0.89–2.21)
Hospitalization or CV-death	243/373 (65.1%)	399/736 (54.2%)	*1.27 (1.06–1.53)*

Significant adjusted hazard ratios are marked in italics

*Adjusted for: age, BMI, history of prior stroke, coronary artery disease or prior revascularization procedure, AF subtype, eGFR (CKD-EPI) and use of ACEI-ARB, OAC and rate control medication after discharge

**Competing-risks regression analysis (Fine and Gray 1999) was performed with all-cause death addressed as a competing risk

DM: diabetes mellitus; HR: hazard ratio; AF: atrial fibrillation; CV: cardiovascular; HF: heart failure

**Fig. 1:**
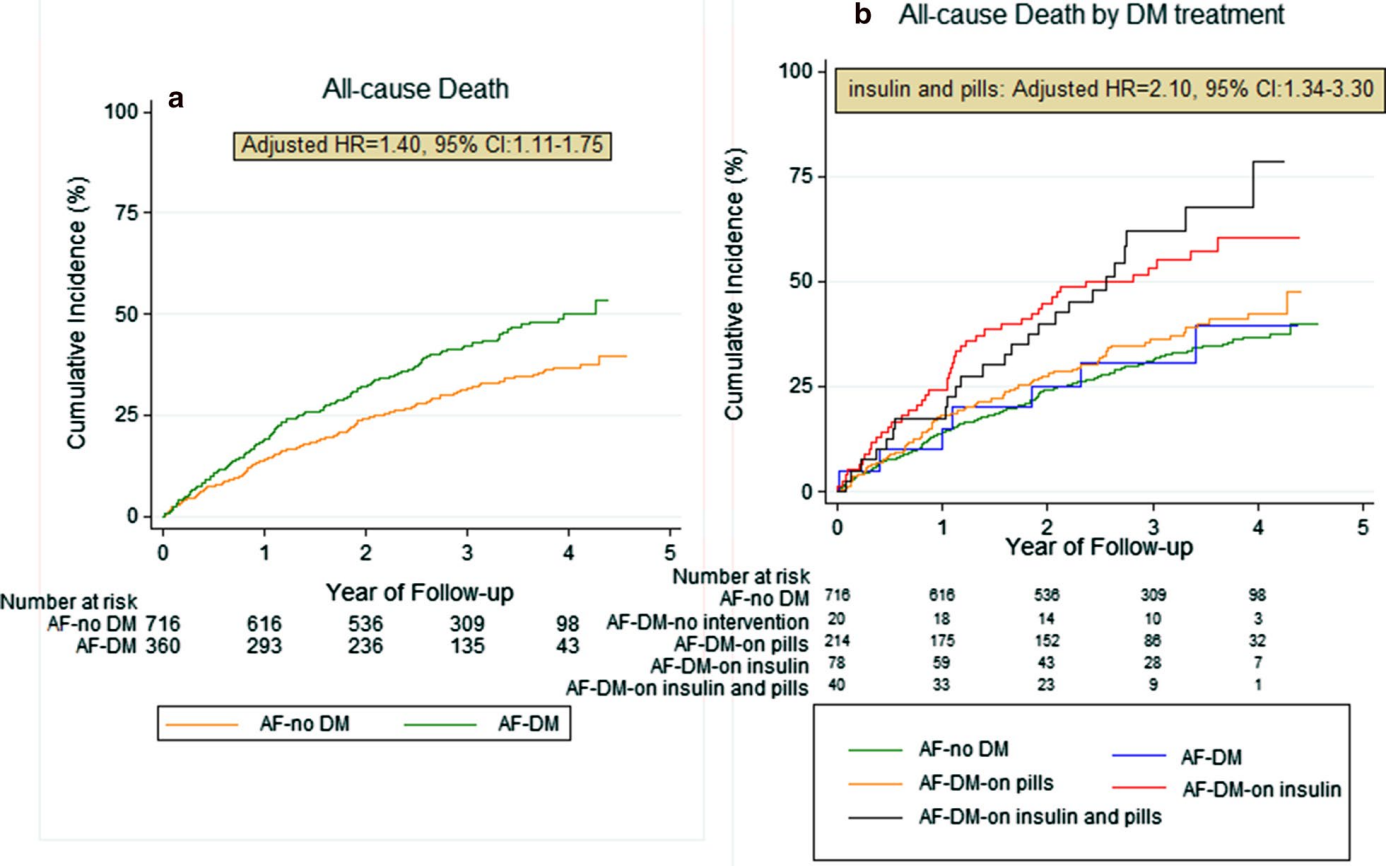
All-cause mortality assessed in terms of: **a** presence or absence of DM and **b** intervention or treatment of DM (Kaplan–Meier analysis). aHR: adjusted hazard ratio; AF: atrial fibrillation; CI: confidence interval; DM: diabetes mellitus

Patients with AF and comorbid DM had worse survival rate compared with those without DM (all-cause death: HR = 1.50, 95% CI 1.20–1.86, p < 0.001) (Fig. 1). Moreover, patients with DM had a significantly higher risk for CV death (HR = 1.56, 95% CI 1.21–2.02), cardiac arrest (HR = 1.94, 95% CI 1.41–2.67), death due to HF (HR = 1.53, 95% CI 1.06–2.19), stroke (HR = 1.92, 95% CI 1.12–3.27), and the composite outcome of CV-death or hospitalization (HR = 1.37, 95% CI 1.17–1.61).

**Fig. 2:**
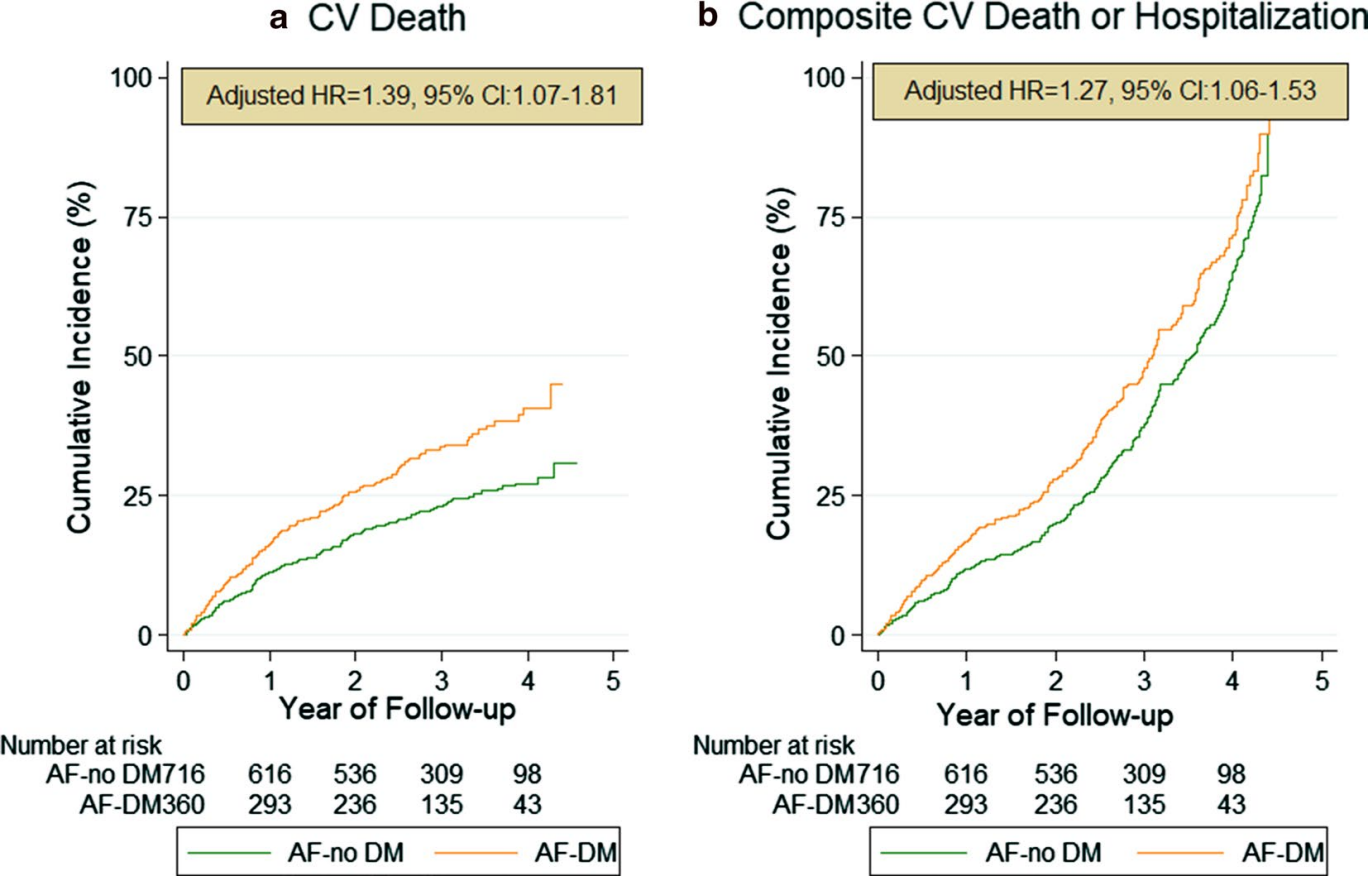
Kaplan–Meier analysis on secondary outcomes: **a** Cardiovascular (CV) death and **b** CV death along with any re-hospitalization during follow up in AF patients with comorbid DM. aHR: adjusted hazard ratio; AF: atrial fibrillation; DM: diabetes mellitus; CV: cardiovascular; CI: confidence interval

**Fig. 3:**
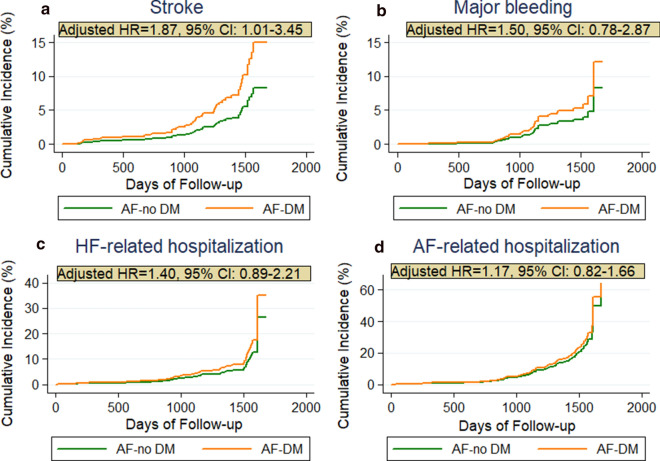
Cumulative incidence curves adjusted for competing risk of death by the presence of DM. The cumulative risk for **a** stroke, **b** major bleeding, **c** HF-, and **d** AF-related hospitalization during the follow-up. aHR: adjusted hazard ratio; AF: atrial fibrillation; CI: confidence interval; DM: diabetes mellitus; HF: heart failure

After adjusting for potential covariates, DM remained significant for predicting all-cause death (aHR = 1.44, 95% CI 1.12–1.85), CV-death (aHR = 1.44, 95% CI 1.08–1.93), cardiac arrest (aHR = 1.73, 95% CI 1.19–2.52), stroke (aHR = 1.87, 95% CI 1.01–3.45) and the composite outcome of CV-death or hospitalization (aHR = 1.28, 95% CI 1.06–1.54) during follow-up (Fig. 2). Both univariable and multivariable competing-risk regression analyses on cumulative incidence of major bleeding events, AF- and HF-related hospitalizations did not yield significantly higher hazard for those events in patients with DM than in those without DM (Fig. 3).

The Kaplan–Meier curves (Fig. 1b) demonstrated difference in the survival rates according to the mode of DM treatment (p < 0.001). Specifically, DM patients under both insulin and oral medication had higher mortality rates during follow-up (aHR = 2.06, 95% CI 1.32–3.23) than those solely on insulin injection, whereas patients with no pharmaceutical intervention or just lifestyle measures had the best prognosis after discharge.

**Fig. 4:**
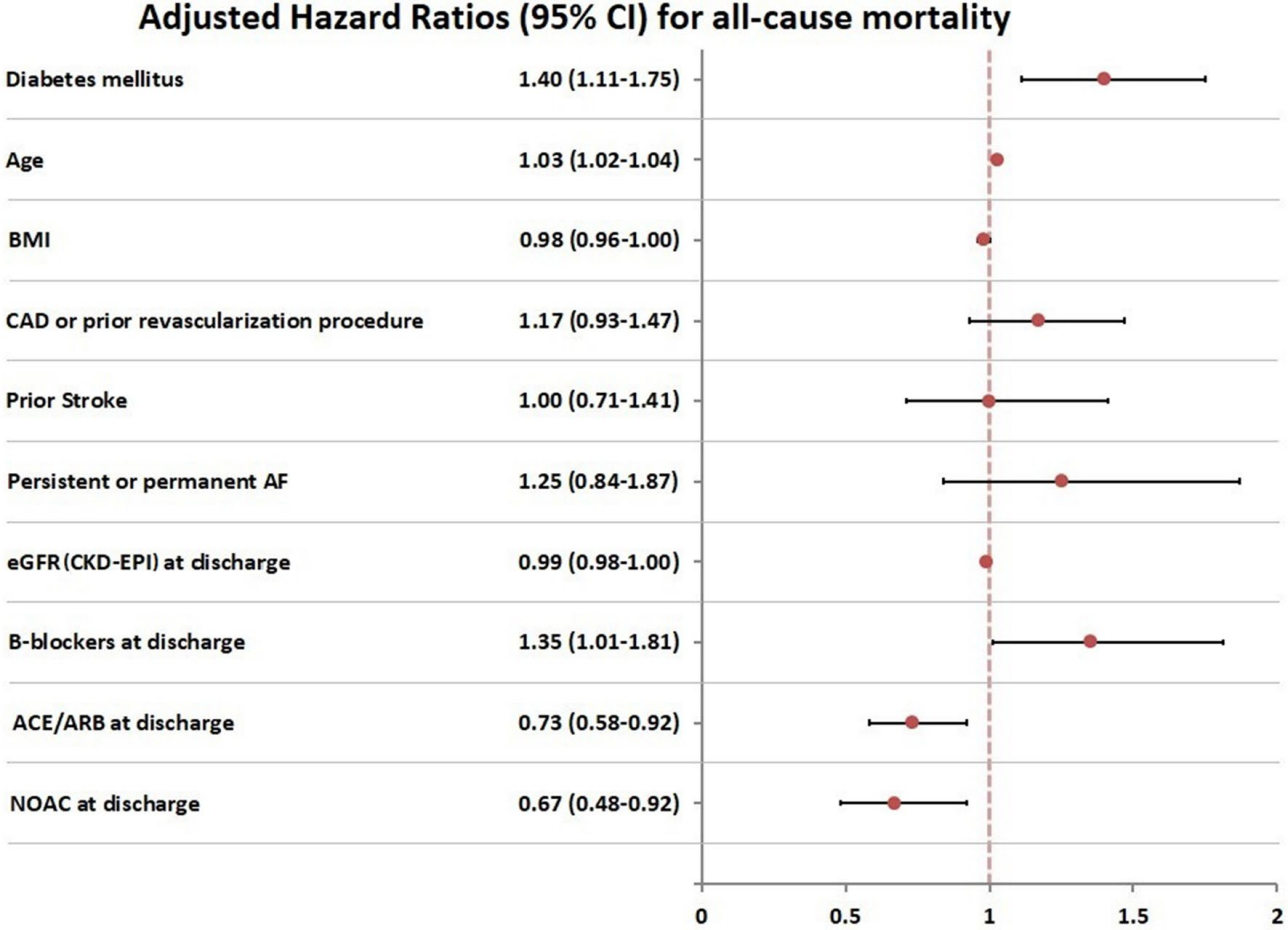
Major subgroup analysis of the primary outcome (all-cause mortality): Predictors of all-cause mortality in the MISOAC-AF patients (Cox multivariable analysis model). Diabetes mellitus maintained statistical significance even after adjustment for confounders (AF subtype, age, BMI, history of prior stroke, coronary artery disease or prior revascularization procedure, eGFR, use of oral anticoagulant(s), ACEI-ARB and rate control medication after discharge). AF: atrial fibrillation; ACE/ARB: angiotensin converting enzyme/angiotensin-receptor blockers; BMI: body mass index; CAD: coronary artery disease; CI: confidence interval; CKD-EPI: Chronic Kidney Disease Epidemiology Collaboration, eGFR: estimated glomerular filtration rate; NOAC: Non-Vitamin K antagonist oral anticoagulant

Our multivariable Cox regression models identified the following predictors of survival as important to AF patients, apart from the presence of DM: age, eGFR, and the use of rate control (b-blocker or/and digoxin), ACEI-ARB and NOAC medication after discharge (p value < 0.05) (Fig. 4).

**Fig. 5:**
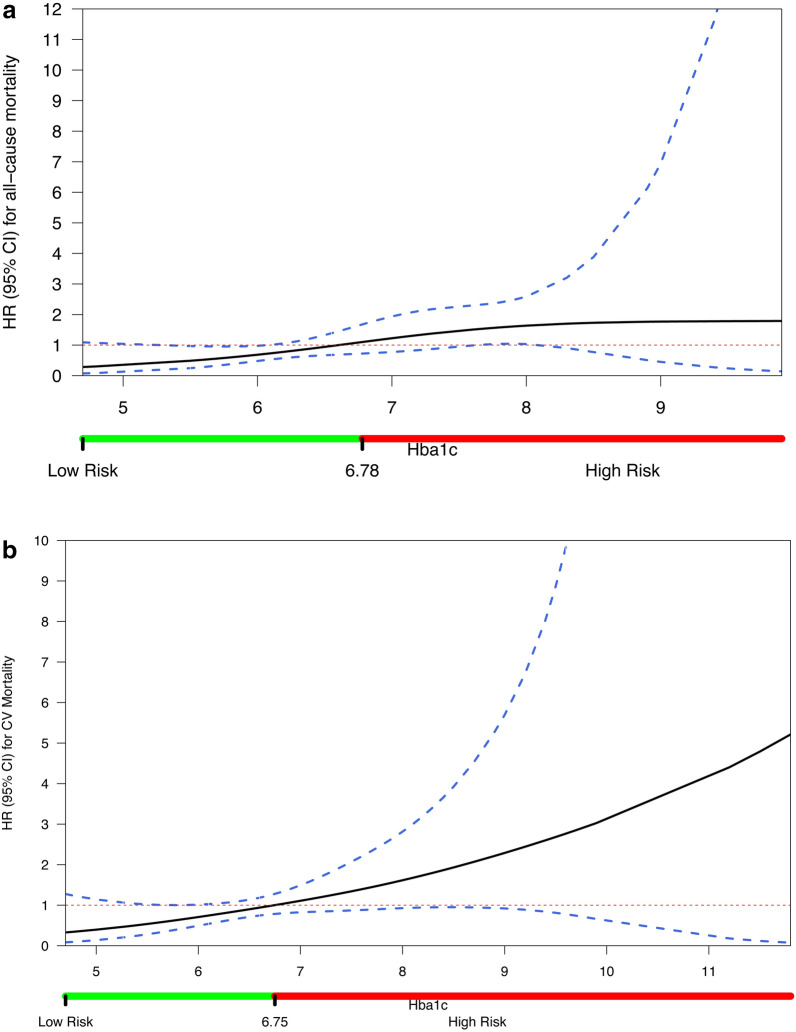
Spline curves correlating **a** all-cause and **b** cardiovascular mortality with HbA1c levels. Dashed lines are 95% confidence intervals and bars below the x-axes represent the subsequently defined glycemic control zones (Low Risk: green HbA1c levels, and High Risk: red HbA1c levels), based on the HbA1c value, in which HR becomes equal to 1. The HRs were adjusted for age, gender, body-mass index, history of dyslipidemia, smoking history, alcohol consumption history, NT-pro-BNP, CRP, eGFR and hemoglobin stages at discharge. Adjusted HRs are displayed on the y-axes. eGFR: estimated glomerular filtration rate; HR: hazard ratio; CRP: C-reactive protein; NT-pro-BNP: N-terminal pro b-type natriuretic peptide

Glycemic status affected mortality, with a 1% increase in HbA1c corresponding to higher all-cause mortality rates (aHR: 1.72, 95% CI 1.13–2.61) and CV mortality rates (aHR: 1.81, 95% CI 1.16–2.82). However, HbA1c levels, as a continuous variable, did not significantly predict hospitalization (aHR: 0.81, 95% CI 0.54–1.23) or stroke incidence (aHR: 0.31, 95% CI 0.06–1.66) during follow-up. Multivariable-adjusted restricted cubic spline analysis (Fig. 5a) suggested an almost linear association between HbA1c levels and the risk for all-cause death, in which an HbA1c value of 6.6% corresponded to aHR of 1. The risk for all-cause mortality was significantly lower at HbA1c levels less than 6.2%. Despite the existing trend towards increased mortality in HbA1c > 6.6%, only HbA1c levels 7.6–8.2% were independently associated with an increased risk of all-cause death. In the spline curve analysis on CV mortality (Fig. 5b) the overall shape of the curve remained linear. This curve had an even more positive slope than the corresponding for all-cause mortality, but the only statistically significant correlation depicted was the lower CV mortality risk (aHR < 1) in the HbA1c range of 5.6–6.2%.

## Discussion

This post hoc analysis of a randomized clinical trial based on an unselected hospitalized population with AF showed that patients with concomitant DM exhibited higher mortality and morbidity rates and suffered more CV events compared to those without DM. To our knowledge, this is the first study to show that chronic glycemic status, represented along the continuum of HbA1c values, considerably predicted all-cause and CV death among patients with AF. HBA1c levels lower than 6.2% were independently associated with decreased risk of death.

DM was present in 1 out of 3 patients and this DM frequency ranks among the highest ones encountered in cohorts with AF patients, since only hospitalized patients were enrolled and not outpatients. The frequency of DM in our study population is almost equal to the one in the large ORBIT-AF registry [6]. The magnitude of association may differ between DM and each subtype of AF, with persistent/permanent AF being most prevalent in our diabetic population. This was ascertained by other studies, such as EORP-AF [7]. Our results corroborate previous observations indicating that patients with AF and comorbid DM are older, more obese, and have a higher frequency of concomitant comorbidities, such as hypertension and impaired renal function than the non-diabetic ones [7, 13]. Patients discharged with comorbid DM were also more likely to suffer from vascular or coronary artery disease, which is plausible due to DM being a major risk factor linked with these diseases [14, 15].

The intimate relationship of AF and DM is already known [16, 17] and the presence of DM is an established marker of worse prognosis, exerting negative impact on quality of life and increasing hospitalization rates in AF patients [18, 19]. Similar to previous studies [20], CV deaths were the most prevalent cause (more than 70% of all causes) of death in both diabetic and non-diabetic participants. Sudden cardiac death and HF-related mortality were the 2 main causes of death in the entire population, with diabetic individuals being at increased risk for suffering those events. Diabetic subjects had a 1.4-fold higher risk for all-cause death or CV death than the non-DM AF population, as well as 1.3-fold higher risk for the composite outcome of CV death or hospitalization during follow-up. These findings concur with those of the EORP-AF and ORBIT-AF studies, which demonstrated a nearly twofold higher risk of mortality in AF patients with comorbid DM, as compared to those without DM [6, 7].

Additionally, DM patients under both insulin and oral medication had worse prognosis than those solely on diet or lifestyle measures, which is logically paralleled to the severity of DM encountered in the respective groups and confounding comorbidities. This finding accords with recent studies showing that patients under insulin-treatment were more likely to suffer thromboembolic events [21–23]. However, the present analysis showed that a higher risk for death or stroke is not confined only to insulin-treated diabetic patients, but concerns the entire diabetic population, regardless of their treatment.

Competing-risk regression analysis showed that patients with DM did not exhibit a significant increase in hospitalizations for AF or HF related complications, in comparison with those without DM. This is in contrast to similar studies, in which DM significantly increased the long-term risk of hospitalization in patients with AF [24, 25]. The analysis on major bleeding events did not correlate with the increased risk for bleeding in DM implied by the HAS-BLED score, possibly due to the sparse events. This may explain the inconsistency with other recent studies [4, 20].

Besides the current study, DM and especially long-term DM has been correlated with higher hazard for stroke in many recent studies [5, 26–29]. However, the glycemic status of these patients seems to be the parameter determining their risk of stroke [30], especially when DM lasts less than 10 years [31]. According to Chan et al. risk of stroke is significantly increased once HbA1c levels exceeded 6.5% in both diabetic and non-diabetic population with AF [32]. This correlation was not mirrored in our analysis, since higher HbA1c levels did not constitute an independent parameter for higher stroke incidence during the follow-up.

It is hypothesized that in AF with DM, the regulation of blood glucose levels could reduce the risk of mortality and adverse CV events. The association of poorly regulated blood glucose levels (HbA1c ≥ 8%) with more frequent adverse outcomes is already established in diabetic populations without AF [33], whereas the present study is the first one to document a linear and positive association between the risk of CV or all-cause mortality and increases in HbA1c values in patients with AF and DM. Other observational studies on diabetic populations have suggested that both low and high HbA1c levels are associated with an increased risk of all-cause mortality (J or U shaped curves) [33, 34]. The observation of increased mortality in extremely low HbA1c levels could not be ascertained in our analysis, maybe due to the limited number of patients with such values. In our study, lower HbA1c levels below a threshold of 6.2% were associated with lower risks of death. Additionally, despite the trend for increased mortality for HbA1c levels above 6.6% values, only values between 7.6 and 8.2% reached statistical significance in showing an increased risk of all-cause mortality.

Therefore, clinicians dealing with patients with AF and DM should view HbA1c levels as an indirect marker of long-term macrovascular risk with low intraindividual variability. Further research is requisite to bolster the prognostic value of HbA1c measurement in this subpopulation and prove whether specific intervention with HbA1c-targeted glycemic control can effectively improve survival. In spite of the guidelines available for the management of DM and AF patients, few work is representative of the unique AF-DM comorbidity [35]. Team work from experts across specialties, including cardiology and endocrinology, is needed to encapsulate the optimal approach for prevention and management of this dual comorbidity.

### Limitations

This study is subject to several limitations. Firstly, DM status was evaluated only at enrollment and we did not ascertain possible new cases arising during the course of follow-up. It is also possible to have some cases of undiagnosed DM at patient enrollment, since oral glucose tolerance test was not performed in individuals not fulfilling the DM classification criteria. Further, a follow-up of 2.6 years potentially failed to depict some of the long-term effects of DM upon the assessed outcomes, whereas the onset, the type and total duration of DM was not coded in our database. We also note the unavailability of HbA1c values for the majority of the non-diabetic study participants and the reliance on a single HbA1c measurement at baseline for the subsequent evaluation of hazard for adverse events during the follow-up period. Moreover, we cannot exclude potential misclassification in registered causes of death and coding inaccuracies, whereas another unavoidable simplification was the classification of some variables (e.g. hypertension) in a merely binary fashion. Notwithstanding the fact that we adjusted for the majority of possible confounders, residual confounding may always exist, owing to the observational nature of our investigation. Additionally, this study lacked a control group of non-AF patients with DM, and therefore we are not able to investigate the influence of AF presence in the DM prognosis. We have also not extracted evidence about novel classes of antidiabetic drugs with potential cardiovascular benefit for AF patients with DM. Lastly, we should not discount the fact that all patients were enrolled in a single institution, which potentially limits the generalizability of our results.

## Conclusions

In this cohort of patients hospitalized with a primary or secondary diagnosis of AF, presence of DM was associated with an almost 1.5-fold increased risk of all-cause death, CV death, sudden cardiac death or the composite outcome of hospitalization or CV death and a twofold increased risk of stroke after patients’ discharge. Our findings suggest a benefit from optimal blood glucose regulation in these patients, given that a threshold below 6.2% was an independent prognostic indicator of reduced mortality. Future studies are warranted to confirm the generalizability of our findings, shed light on the conundrum of interactions between AF and DM, and delineate specific management strategies for fighting the AF and DM epidemic.

## Data Availability

Data are available from George Giannakoulas (e-mail: ggiannakoulas@auth.gr) upon reasonable request and with permission of AHEPA University Hospital.

## Abbreviations

AF: Atrial fibrillation
(a)HR: (Adjusted) hazard ratio
BMI: Body mass index
CI: Confidence interval
CKD-EPI: Chronic Kidney Disease Epidemiology Collaboration
DM: Diabetes mellitus
eGFR: Estimated glomerular filtration rate
HF: Heart failure
NOAC: Non-vitamin K antagonist oral anticoagulants
NT-proBNP: N-terminal pro b-type natriuretic peptide
OAC: Oral anticoagulant
VKA: Vitamin K antagonists
